# Negative Pressure Wound Therapy for the Prevention of Wound Complications After Hepatopancreatobiliary Surgery: A Systematic Review and Meta‐Analysis

**DOI:** 10.1002/hsr2.72749

**Published:** 2026-07-04

**Authors:** Xiaofei Ma, Lin Li, Adriana C. Panayi, Liming Zhong, Lin Luo, Ziyu Chen, Mengfan Wu

**Affiliations:** ^1^ Department of Hepatobiliary and Pancreatic Surgery Peking University Shenzhen Hospital Shenzhen Guangdong People's Republic of China; ^2^ Department of Oral and Maxillofacial Surgery Charité—Universitätsmedizin Berlin, Corporate Member of Freie Universität Berlin, Humboldt‐Universität zu Berlin, and Berlin Institute of Health Berlin Germany; ^3^ Department of Plastic Surgery Peking University Shenzhen Hospital Shenzhen Guangdong People's Republic of China; ^4^ Department of Bone and Joint Surgery Peking University Shenzhen Hospital, Shenzhen Peking University—The Hong Kong University of Science and Technology Medical Center Shenzhen Guangdong People's Republic of China

**Keywords:** HPB surgery, negative pressure wound therapy, surgical site infections, would healing

## Abstract

**Background and Aims:**

Hepatopancreatobiliary (HPB) surgeries, such as hepatic resection and pancreaticoduodenectomy, are associated with a high incidence of postoperative wound complications, particularly surgical site infections (SSIs). Negative pressure wound therapy (NPWT) has been proposed to reduce such complications, but its effectiveness in HPB surgery remains uncertain. This systematic review and meta‐analysis aimed to evaluate the association between NPWT and postoperative wound complications following HPB procedures.

**Methods:**

We systematically searched PubMed, EMBASE, Cochrane Library, Web of Science, and Scopus through August 17, 2024 for randomized controlled trials (RCTs) and observational studies comparing NPWT with standard surgical dressing (SSD) after HPB surgery. Primary outcomes were overall, superficial, deep, and organ‐space SSIs. Secondary outcomes included seroma, hematoma, wound dehiscence, and hospital readmission. Risk ratios (RRs) with 95% confidence intervals (CIs) were pooled using a random‐effects model.

**Results:**

Eleven studies (7 RCTs, 4 observational; *n* = 1566) were included. Pooled analyses suggested that NPWT was associated with a lower risk of overall SSI compared with SSD (RR = 0.58, 95% CI = 0.45–0.75, *p* < 0.0001). When restricted to RCTs, the effect size was smaller and only approached statistical significance (RR = 0.70, *p* = 0.05). Superficial SSI (sSSI) rates were lower in the NPWT group, although this finding was not maintained in sensitivity analyses. No significant differences in deep or organ‐space infections, or other secondary outcomes. Subgroup analyses suggested a greater potential benefit in pancreatectomy cases (RR = 0.61, *p* = 0.001). PICO devices were associated with reduced SSI risk, whereas PREVENA systems showed no statistically significant association.

**Conclusions:**

NPWT may reduce postoperative SSIs following HPB surgeries, particularly in patients undergoing pancreatectomy. However, the observed benefit was less robust in RCT‐only analyses, and evidence for liver surgery remains limited. Further high‐quality RCTs are needed to validate these findings.

## 1 Introduction

Hepatopancreatobiliary (HPB) surgeries, including complex procedures such as hepatic resection and pancreaticoduodenectomy (PD), are associated with a high risk of postoperative wound complications [1, 2]. These complications include surgical site infections (SSIs), seromas, hematomas, and wound dehiscence. Such complications lead to increased patient discomfort, delays in adjuvant therapies, higher financial burdens, and a reduced quality of life. SSIs rates as high as 25% after PD and 23% following hepatic and pancreatic resections have been reported [3, 4]. Postoperative wound complications also increase nursing workload, prolong hospital stays, and contribute substantially to healthcare resource utilization and postoperative care demands [2]. In the current healthcare environment, enhanced recovery protocols and value‐based perioperative care are increasingly emphasized [5]. Prevention of postoperative wound complications has become an important multidisciplinary priority. Various strategies have been explored to reduce wound complications including wound irrigation, pulse lavage, open‐ or closed‐suction drainage, antimicrobial‐coated sutures, and delayed primary closure [6, 7, 8, 9, 10, 11]. However, many of these approaches lack robust evidence of significantly reducing SSI rates. This leaves substantial gaps in the identification of effective, evidence‐based interventions [12].

Negative Pressure Wound Therapy (NPWT) is currently endorsed by the World Health Organization SSI Guidelines for high‐risk surgical wounds [13, 14, 15]. This endorsement is due to its mechanisms, which include improving tissue perfusion, reducing edema, and promoting exudate removal [16]. While NPWT has shown positive outcomes in various surgical contexts, its specific efficacy in HPB surgeries remains controversial. Wells et al. performed a meta‐analysis showing that NPWT significantly reduced the incidence of SSIs after laparotomy [17], and Zhang et al. reported an 11‐point reduction in SSIs for patients undergoing PD [18]. However, Ren et al. meta‐analysis observed only a slight decrease in hospital readmissions, with no significant effects on superficial or deep SSIs, seromas, or hematomas following HPB surgeries [19]. Additionally, Lenet's meta‐analysis concluded that NPWT does not significantly lower the risk of SSIs in patients undergoing pancreatectomy [20]. Importantly, previous meta‐analyses were limited by small sample sizes, inconsistent inclusion criteria, and substantial clinical heterogeneity, particularly regarding surgical type, NPWT protocols, and study design.

To address the ongoing uncertainty surrounding the use of NPWT in HPB surgeries, this systematic review and meta‐analysis aims to rigorously assess its efficacy in reducing postoperative wound complications. By synthesizing evidence from both randomized and real‐world clinical studies, this study seeks to clarify the current evidence base and evaluate whether NPWT may represent a clinically meaningful strategy for reducing postoperative wound complications in high‐risk HPB patients. Ultimately, this study intends to provide robust evidence that will guide clinical decision‐making. Such evidence will help surgeons and healthcare providers adopt evidence‐based strategies to optimize postoperative care and improve patient outcomes after HPB surgeries.

## 2 Methods

### 2.1 Search Strategy

This systematic review and meta‐analysis was conducted in accordance with the Preferred Reporting Items for Systematic Reviews and Meta‐Analyses (PRISMA) guidelines [21] and registered on PROSPERO (Registration Number: CRD42024564229). We performed a comprehensive literature search of the following databases: PUBMED, EMBASE, Cochrane Library, Web of Science, and Scopus databases from inception to August 17, 2024, for studies published in the English language. The search strategy combined terms related to “negative pressure wound therapy,” “wound complications,” and “hepatopancreatobiliary surgery.” The complete search strategy for each database is provided in Supplementary Materials 1.

### 2.2 Inclusion and Exclusion Criteria

Studies were included if they focused on patients undergoing HPB surgery and evaluated the use of NPWT applied to surgical incisions postoperatively. Eligible studies compared NPWT with conventional wound care or no NPWT and reported outcomes related to wound complications, including SSIs, seroma, hematoma, readmission, and other adverse events. We considered randomized controlled trials (RCTs), prospective cohort studies, and retrospective cohort studies for inclusion. Studies were excluded if they involved animal models, were case reports or review articles, or lacked an appropriate comparator/control group.

### 2.3 Data Extraction and Quality Assessment

Two independent reviewers (X.M. and L.L.) screened titles and abstracts for eligibility and subsequently reviewed the full texts of potentially relevant studies. Discrepancies were resolved through discussion or by consulting a third reviewer (M.W.). For each included study, the following data were extracted: study characteristics (author, year of publication, country, study design), patient demographics, type of HPB surgery, details of NPWT application, and outcomes of interest. Quality assessment was performed independently by two reviewers (X.M. and L.L.) using the Cochrane Risk of Bias (RoB2) Tool [22] for RCTs and the Newcastle‐Ottawa Scale (NOS) [23] for observational studies.

### 2.4 Statistical Analysis

Since all outcomes were dichotomous, pooled effect sizes were calculated using risk ratios (RRs) with corresponding 95% confidence intervals (CIs). Heterogeneity was assessed using the *I* ^2^ statistic and Cochran's *Q* test, with significant heterogeneity defined as an *I* ^2^ > 50% or *p* < 0.10. Meta‐analysis was performed using a random‐effects model to account for potential clinical and methodological heterogeneity among studies.

### 2.5 Subgroup Analysis

We conducted subgroup analyses to assess the impact of NPWT on SSIs by stratifying studies based on sample size, performing a focused analysis of pancreatectomy outcomes, and evaluating different NPWT devices.

### 2.6 Sensitivity Analysis and Publication Bias

We conducted sensitivity analyses by excluding studies at high risk of bias and evaluating the impact on the pooled estimates. Publication bias was assessed by visual inspection of funnel plots, Egger's regression test, and nonparametric trim‐and‐fill analysis when applicable.

### 2.7 Certainty of Evidence Assessment

The certainty of evidence for the primary outcomes was assessed using the Grading of Recommendations Assessment, Development and Evaluation (GRADE) framework. Evidence quality was evaluated across the domains of risk of bias, inconsistency, indirectness, imprecision, and publication bias. The certainty of evidence was categorized as high, moderate, low, or very low.

## 3 Results

### 3.1 Study Selection

**Figure 1:**
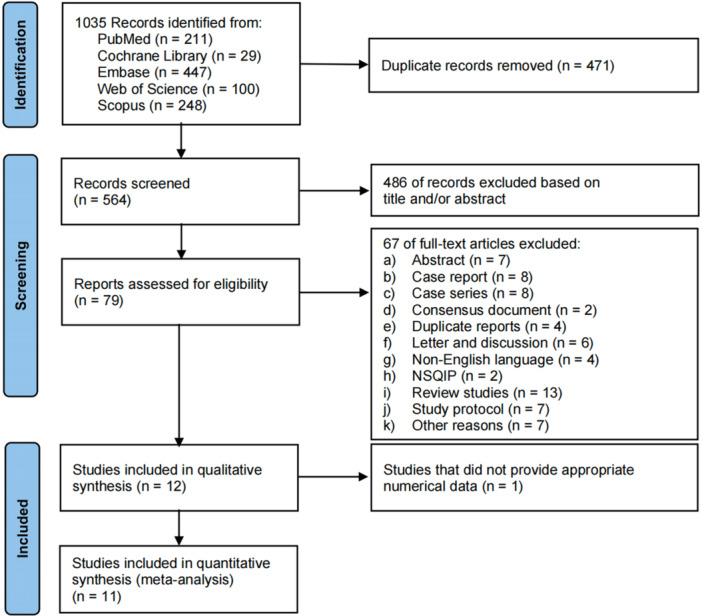
PRISMA flowchart detailing literature search and screening strategy.

The initial literature search identified a total of 1035 unique records. After removing duplicates, 564 studies remained for title and abstract screening. Subsequently, 486 studies were excluded for not meeting the inclusion criteria, leaving 79 full‐text articles for detailed evaluation. Following full‐text screening, 67 studies were excluded for reasons such as ineligible study design (e.g., abstract, case reports, reviews), lack of an appropriate control group, non‐English language, duplicate reports, or other reasons, leaving 12 studies in the final systematic review. After excluding one study that did not provide appropriate numerical data [24], 11 studies were included in the final meta‐analysis [25, 26, 27, 28, 29, 30, 31, 32, 33, 34, 35]. The PRISMA flow diagram summarizing the study selection process is presented in Figure 1.

### 3.2 Study Characteristics

The final set of included studies comprised seven RCTs and four observational studies, comprising of a total of 1566 patients undergoing various HPB procedures, with 667 patients receiving NPWT (NPWT group) and 899 patients receiving standard surgical dressing (SSD) treatment postoperatively. The studies varied in design, sample size, and geographic location, and were conducted in North America, Europe, and Asia. The types of HPB surgeries analyzed included hepatic resections, pancreatectomy (pancreaticoduodenectomies, distal or subtotal pancreatectomy, total pancreatectomy), and liver transplantation. The details of NPWT application, such as timing, duration, and specific device settings, differed across studies. A detailed summary of the study characteristics is provided in Table 1.

**Table 1:** Characteristics of the included studies.

No.	First author	Year	Country	Study type	Number of patients (NPWT/Control)	Surgery	NPWT details	Control
1	Andrianello	2021	Italy	RCT	95 (46/49)	Major pancreatic resections (PD, total pancreatectomy or gastro‐jejunal and biliary bypassd)	PICO portable NPWT device (Smith & Nephew healthcare), changed POD3, removed POD7	Sterile gauze, uncovered POD3, recovered with SSD
2	Burkhart	2017	USA	Prospective	394 (120/274)	PD	Kinetic concepts VAC (KCI), −125 mmHg, removed POD5	SSD, removed POD2
3	Ceppa	2023	USA	RCT	138 (63/75)	HPB surgery, CRS	Prevena Incision Management System (3 M Health Care), removed POD2‐7	SSD, removed POD2
4	Greene	2023	Canada	Retrospective cohort study	175 (61/114)	PD	Prevena Incision Management System (Acelity), −125 mmHg, removed POD7	SSD, removed POD2
5	Gupta	2017	USA	Retrospective cohort study	61 (25/36)	PD (Whipple)	No report, used for 7–10 days	Traditional dressing
6	Javed	2019	USA	RCT	123 (62/61)	PD	Prevena Peel & Place Dressing (Acelity), −125 mmHg, removed POD5	SSD, removed POD2
7	Kuncewitch	2019	USA	RCT	73 (36/37)	Pancreatectomy (PD, distal or subtotal pancreatectomy, total pancreatectomy)	Kinetic concepts VAC (KCI), −125 mmHg, removed POD4	SSD, removed POD4
8	Lawrence	2019	USA	Prospective Cohort Study	300 (150/150)	PD	PICO System (Smith & Nephew), removed POD7	SSD
9	Lopez‐Lopez	2023	Spain	RCT	108 (54/54)	LT	PICO portable NPWT device (Smith & Nephew healthcare), −80 mmHg, removed POD5‐7	SSD
10	O'Neill	2020	USA	RCT	40 (20/20)	Hepatic or pancreatic resections	PICO System (Smith & Nephew), −50 ~ −175 mmHg, removed POD7	Sterile island dressing
11	Shen	2016	USA	RCT	265 (132/133)	open resection of intra‐abdominalneoplasms: gastrointestinal, pancreas, and peritoneal surface malignancy	No report, −125 mmHg, removed POD4	SSD, removed POD4

Abbreviations: CRS, colorectal surgery; HPB, hepatopancreatobiliary; LT, liver transplantation; NPWT, negative pressure wound therapy; PD, pancreaticoduodenectomy; POD, postoperative day; RCT, randomized controlled trials; SSD, standard surgical dressing.

### 3.3 Risk of Bias

Risk of bias of the included studies was assessed using the RoB2 tool for RCTs and the NOS for observational studies, as detailed in Supplementary Materials 2. Overall, most RCTs were rated as having a low risk of bias, particularly in the domains of missing outcome data and selection of the reported result. However, some trials presented concerns related to deviations from the intended interventions and randomization processes. For observational studies, the quality was generally moderate to high. Most studies were rated well, with only Gupta's study showing limitations [28], primarily in the areas of selection and outcome assessment criteria.

### 3.4 Study Outcomes

#### 3.4.1 Primary Outcome: Surgical Site Infections (SSI)

**Figure 2:**
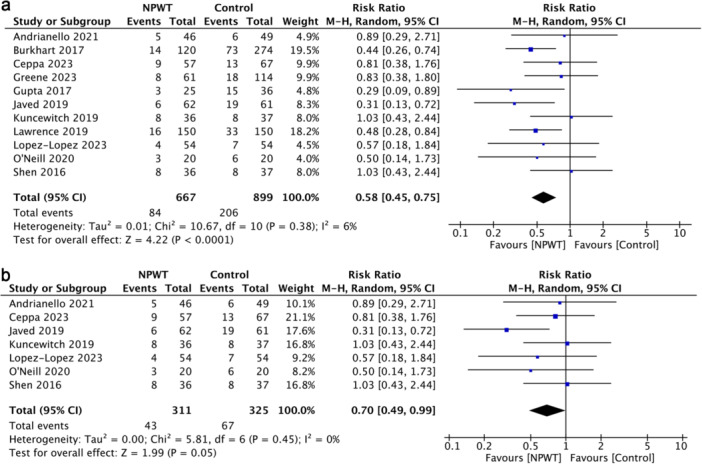
Forest plots comparing NPWT with SSD for the incidence of SSI. (a) Overall SSI including all eligible studies; (b) SSI analysis restricted to RCTs.

The primary outcome was the incidence of SSIs, including superficial SSI (sSSI), deep SSI (dSSI), and organ space infection (OSI). In the pooled analysis combining RCTs and observational studies, NPWT was associated with a lower risk of overall SSI compared to SSD (RR = 0.58, 95% CI = [0.45, 0.75], *p* < 0.0001; Heterogeneity *I* ^2^ = 6%, *p* = 0.38; Figure 2a).

However, when analyses were restricted to RCTs, the effect estimate was attenuated and only approached statistical significance (RR = 0.70, 95% CI = [0.49, 0.99], *p* = 0.05; Heterogeneity *I* ^2^ = 0%, *p* = 0.45; Figure 2b). These findings suggest that the overall pooled effect may have been influenced by the inclusion of observational studies.

**Figure 3:**
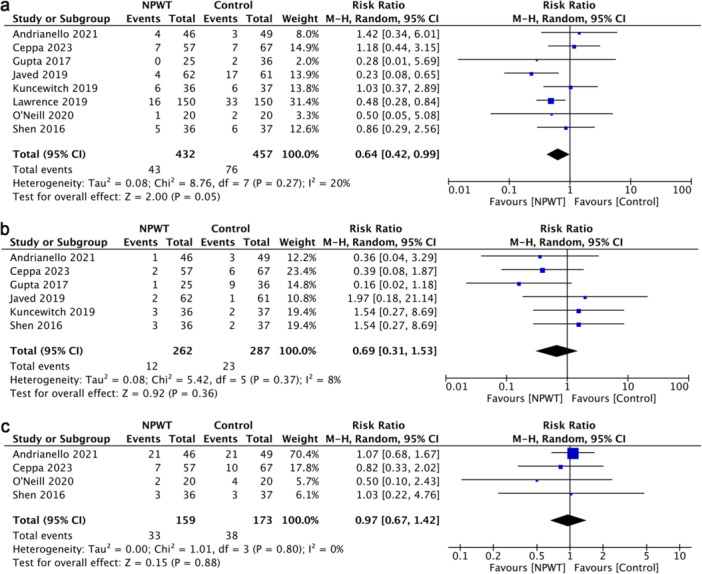
Forest plots comparing NPWT with SSD for specific types of SSI. (a) Superficial SSI (sSSI); (b) deep SSI (dSSI); and (c) organ‐space infection (OSI).

The incidence of sSSI was lower in the NPWT group, with the result approaching statistical significance (RR = 0.64, 95% CI = [0.42, 0.99], *p* = 0.05; Heterogeneity *I* ^2^ = 20%, *p* = 0.27; Figure 3a), while the reduction in dSSI and OSI did not reach statistical significance (RR = 0.69, 95% CI = [0.31, 1.53], *p* = 0.36; Heterogeneity *I* ^2^ = 8%, *p* = 0.37; Figure 3b; RR = 0.97, 95% CI = [0.67, 1.42], *p* = 0.88; Heterogeneity *I* ^2^ = 0%, *p* = 0.80; Figure 3c).

#### 3.4.2 Secondary Outcomes

**Figure 4:**
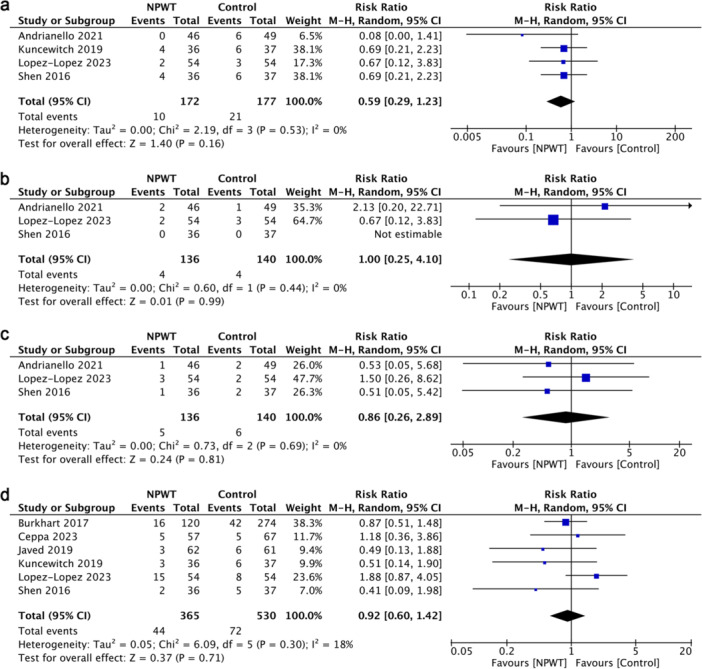
Forest plots comparing NPWT with SSD for secondary postoperative wound outcomes, including (a) seroma, (b) hematoma, (c) wound dehiscence, and (d) hospital readmission.

The secondary outcomes analysis showed no statistically significant differences between the NPWT and control groups for seroma (RR = 0.59, 95% CI = [0.29, 1.23], *p* = 0.16; Heterogeneity *I* ^2^ = 0%, *p* = 0.53; Figure 4a), hematoma (RR = 1.00, 95% CI = [0.25, 4.10], *p* = 0.99; Heterogeneity *I* ^2^ = 0%, *p* = 0.44; Figure 4b), wound dehiscence (RR = 0.86, 95% CI = [0.26, 2.89], *p* = 0.81; Heterogeneity *I* ^2^ = 0%, *p* = 0.69; Figure 4c), and readmission rates (RR = 0.92, 95% CI = [0.60, 1.42], *p* = 0.71; Heterogeneity *I* ^2^ = 18%, *p* = 0.30; Figure 4d).

### 3.5 Subgroup Analysis

**Figure 5:**
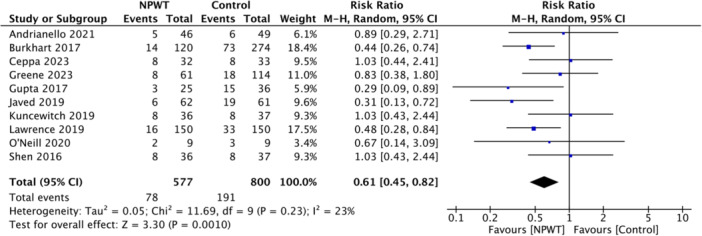
Subgroup analysis evaluating the effect of NPWT versus SSD on SSI in patients undergoing pancreatectomy.

In the subgroup analysis, the effect of NPWT on SSI was stratified by sample size, pancreatectomy, and device type (Table 2). For studies with ≤ 100 patients, the reduction in SSI was not statistically significant (RR = 0.74, 95% CI = [0.46, 1.18], *p* = 0.21; Heterogeneity *I* ^2^ = 8%, *p* = 0.36). Conversely, studies with > 100 patients showed a significant reduction in SSIs (RR = 0.52, 95% CI = [0.39, 0.69], *p* < 0.00001; Heterogeneity *I* ^2^ = 0%, *p* = 0.46). When stratifying by ≤ 150 patients, a moderate effect was observed (RR = 0.64, 95% CI = [0.44, 0.92], *p* = 0.02; Heterogeneity *I* ^2^ = 12%, *p* = 0.33), while studies with > 150 patients exhibiting a more pronounced reduction (RR = 0.52, 95% CI = [0.37, 0.73], *p* = 0.0002; Heterogeneity *I* ^2^ = 0%, *p* = 0.39). For patients undergoing pancreatectomy, NPWT significantly decreased the risk of SSI (RR = 0.61, 95% CI = [0.45, 0.82], *p* = 0.0010; Heterogeneity *I* ^2^ = 23%, *p* = 0.23; Figure 5). For NPWT devices, PICO demonstrated a significant reduction in SSI (RR = 0.54, 95% CI = [0.35, 0.83], *p* = 0.005; Heterogeneity *I* ^2^ = 0%, *p* = 0.82), whereas PREVENA showed a non‐significant effect (RR = 0.61, 95% CI = [0.33, 1.13], *p* = 0.11; Heterogeneity *I* ^2^ = 44%, *p* = 0.17).

**Table 2:** Pooled risk ratios for SSI of subgroup analyses.

Subgroup analyses		No. of studies	RR (M‐H, Random, 95% CI)	*p* value	Heterogeneity	
					*p* value	*I* ^2^ (%)
All studies		11	0.58 [0.45, 0.75]	< 0.0001	0.38	6
Sample size	≤ 100 patients	5	0.74 [0.46, 1.18]	0.21	0.36	8
	> 100 patients	6	0.52 [0.39, 0.69]	< 0.00001	0.46	0
	≤ 150 patients	8	0.64 [0.44, 0.92]	0.02	0.33	12
	> 150 patients	3	0.52 [0.37, 0.73]	0.0002	0.39	0
Pancreatectomy		10	0.61 [0.45, 0.82]	0.0010	0.23	23
Device	PICO	4	0.54 [0.35, 0.83]	0.005	0.82	0
	PREVENA	3	0.61 [0.33, 1.13]	0.11	0.17	44

### 3.6 Sensitivity Analysis

Sensitivity analyses were performed by excluding studies deemed at high risk of bias and reanalyzing the primary outcomes (Table 3). The findings for overall SSIs, dSSIs, and OSIs remained consistent, affirming the robustness of these results. However, the reduction in sSSI no longer achieved statistical significance (RR = 0.66, 95% CI = [0.41, 1.05], *p* = 0.08; Heterogeneity *I* ^2^ = 29%, *p* = 0.20), suggesting a potential impact of study quality on this outcome and emphasizing the need for cautious interpretation regarding the efficacy of NPWT in preventing superficial infections.

**Table 3:** Sensitivity analysis.

Primary Outcomes	No. of studies	RR (M‐H, Random, 95% CI)	*p* value	Heterogeneity	
				*p* value	*I* ^2^ (%)
SSI	10	0.60 [0.47, 0.77]	< 0.0001	0.43	1
sSSI	7	0.66 [0.41, 1.05]	0.08	0.20	29
dSSI	5	0.88 [0.39, 2.02]	0.77	0.57	0
OSI	4	0.97 [0.67, 1.42]	0.88	0.80	0

Abbreviations: dSSI, deep SSI; OSI, organ space infection; RR, risk ratio; SSI, surgical site infection; sSSI, superficial SSI.

### 3.7 Publication Bias

Publication bias assessments (Supplementary Materials 3) were constructed to assess potential publication bias for the analysis of overall SSI, which included 11 studies. Visual inspection of the funnel plot did not demonstrate substantial asymmetry. Egger's regression test did not detect significant publication bias for overall SSI (*p* = 0.525). Nonparametric trim‐and‐fill analysis identified no potentially missing studies, and the pooled effect estimate remained unchanged after adjustment, suggesting relative robustness of the primary findings.

### 3.8 Certainty of Evidence

GRADE assessment demonstrated low‐to‐very‐low certainty of evidence for the primary outcomes (Supplementary Materials 4). The certainty of evidence for overall SSI and the pancreatectomy subgroup was rated as low, mainly due to inclusion of observational studies and indirectness related to procedural heterogeneity. The certainty of evidence for superficial SSI was rated as very low because of imprecision and instability in sensitivity analyses.

## 4 Discussion

### 4.1 Comparison With Previous Meta‐Analyses and Critical Appraisal

This systematic review and meta‐analysis shows that NPWT may be associated with a reduced risk of SSI compared to SSD in HPB surgeries. The pooled analysis demonstrated a 42% relative reduction in overall SSI risk. However, when analyses were restricted to RCTs, the effect estimate was attenuated and only approached statistical significance. This indicates uncertainty regarding the magnitude of benefit. The observed association appeared more evident for sSSI, while reductions in dSSI and OSI did not achieve statistical significance. In addition, the observed reduction in superficial SSI was not maintained after sensitivity analyses excluding high‐risk studies, suggesting limited robustness of this finding. Secondary outcomes—such as seroma, hematoma, wound dehiscence, and readmission rates—showed no statistically significant differences between NPWT and SSD. Subgroup analyses suggested a potentially greater benefit in patients undergoing pancreatectomy, although these findings should be interpreted as exploratory.

Prior meta‐analyses on NPWT's effectiveness in HPB surgeries have yielded inconsistent results, which limitins clinical applicability. For instance, in 2022, a meta‐analysis, which included five RCTs and one observational study. It reported only a slight reduction in hospital readmissions with NPWT but observed no significant impact on sSSI, dSSI, seroma, or hematoma rates [19]. Another meta‐analysis, which included four RCTs and three observational studies, showed that NPWT are effective in reducing the incidence of SSI by 11 points in patients undergoing PD. However, these findings of this previous analyses were complicated by significant clinical and methodological heterogeneity. This leaves uncertainty in conclusions and limiting guidance for clinical practice [18].

Our meta‐analysis expands upon prior research by incorporating a broader range of studies, including the largest number of currently available studies and patients. We included a total of seven RCTs and four observational studies, covering 1566 patients. This expanded dataset provides a broader overview of the available evidence. Nevertheless, the certainty of evidence remains limited by study heterogeneity and the inclusion of observational studies. Observational studies may introduce residual confounding and selection bias, potentially inflating the observed treatment effect. Therefore, the current findings should be interpreted as supportive rather than definitive evidence of NPWT efficacy in HPB surgery.

The pronounced benefit of NPWT in pancreatectomy likely reflects the high‐risk profile of these patients. Wound complications remain a significant burden in patients undergoing pancreatectomy despite targeted interventions and heightened awareness [36]. The incidence of SSI after PD varies from 12% to 51% [37, 38]. This heightened risk is likely attributable to several surgical factors, such as prolonged operative times, high intraoperative transfusion requirements, and an elevated risk of pancreatic anastomotic leakage [39]. Additionally, patients undergoing pancreatectomy often have predisposing baseline factors, including poor preoperative nutritional status, the need for preoperative biliary drainage, or prior neoadjuvant chemotherapy. All of which further increase their susceptibility to wound complications [40]. NPWT may play a critical role in mitigating these risks, given its mechanisms of enhancing tissue perfusion, reducing edema, and removing exudate [41]. A 39% relative reduction in SSI risk was observed in the pancreatectomy subgroup analysis. Nevertheless, subgroup analyses are inherently exploratory and should not be interpreted as conclusive evidence of superiority in a specific surgical population.

Differences between NPWT devices may also have contributed to variability in outcomes. PICO devices demonstrated an association with lower SSI risk, whereas PREVENA systems did not show statistically significant effects. These findings should be interpreted cautiously because the number of studies within each subgroup was limited. Potential explanations for these differences may include variation in device design, portability, exudate handling capacity, negative pressure settings, and duration of therapy. However, the currently available literature lacks standardized NPWT treatment protocols and reporting consistency. This limits the ability to determine whether observed differences are attributable to device‐specific characteristics or study‐level heterogeneity. Therefore, conclusions regarding device superiority cannot currently be established. Future studies using standardized protocols and direct comparative designs are needed to clarify the relative effectiveness of different NPWT systems in HPB surgery.

### 4.2 Strengths and Limitations

This study has several strengths. We included both randomized and observational studies, allowing assessment of NPWT effectiveness across controlled and real‐world clinical settings. Subgroup, sensitivity, and device‐specific analyses were also performed to explore sources of heterogeneity and assess the robustness of findings.

Although our meta‐analysis provides more conclusive evidence, several limitations remain within the included studies. First, substantial clinical heterogeneity existed among the included studies, particularly regarding surgical procedures, patient populations, NPWT protocols, and device types. Most studies focusing on pancreatectomy, whereas the data for hepatic surgeries were sparse, with only three studies including liver procedures, and only two provided extractable hepatic‐specific outcome data. Furthermore, these studies evaluated clinically distinct procedures, including liver transplantation and hepatic resection, which limited the feasibility and validity of pooled subgroup analysis. Consequently, the applicability of the current findings to liver surgery remains uncertain.

Importantly, the overall pooled effect was influenced by the inclusion of observational studies, which may introduce residual confounding and selection bias. Furthermore, several subgroup analyses were based on limited sample sizes and should be interpreted as exploratory. Variability in NPWT devices, pressure settings, and treatment duration also contributed to clinical heterogeneity across studies.

In terms of the review methodology, one key limitation was the potential for language bias, as only studies published in English were included. Although formal publication bias analyses were performed, the relatively small number of included studies may still limit the sensitivity of these assessments. Finally, the exclusion of non‐randomized or non‐controlled studies may have omitted relevant data, though this was a necessary step to maintain the methodological rigor of the analysis.

### 4.3 Clinical Implications

The findings from this meta‐analysis indicate that NPWT may be an effective strategy for reducing SSI rates following HPB surgeries, especially in high‐risk procedures such as pancreatectomy. Given the substantial morbidity and healthcare burden associated with postoperative SSI, selective use of NPWT in carefully chosen high‐risk patients may be clinically reasonable.

However, the current evidence does not support universal application of NPWT across all HPB procedures. The reduced effect size observed in RCT‐only analyses and the limited evidence for liver surgery highlight the need for cautious interpretation. Clinicians should also consider factors such as patient risk profile, surgical complexity, device availability, and cost‐effectiveness when selecting NPWT in postoperative wound management.

### 4.4 Future Research Directions

Future adequately powered RCTs are needed to confirm the magnitude of benefit observed in pooled analyses. They are also needed to determine which patient populations are most likely to benefit from NPWT. Future studies should specifically evaluate NPWT in homogeneous hepatic surgery populations, to clarify whether the benefits observed in pancreatic surgery can be generalized to other HPB procedures. Additionally, future studies should work towards standardizing the NPWT treatment protocol, including its application, duration, and cost‐effectiveness, to enhance clinical implementation. Exploring the mechanisms by which NPWT reduces SSIs, investigating long‐term outcomes, and addressing uncertainties related to secondary outcomes such as seroma, hematoma, and wound dehiscence—where our analysis found no significant differences—should be prioritized in subsequent research efforts.

## 5 Conclusion

This meta‐analysis suggests that NPWT may reduce the risk of overall SSI in HPB surgeries, particularly in patients undergoing pancreatectomy. However, the observed benefit was less robust in analyses restricted to RCTs, and findings for sSSI were not stable in sensitivity analyses. Evidence for liver surgery remains limited, and variability in NPWT protocols and device types contributes to uncertainty in the current evidence base. Further adequately powered randomized trials are needed before routine use can be broadly recommended.

## Author Contributions

**Xiaofei Ma:** data curation, investigation, formal analysis, writing – original draft, visualization. **Lin Li:** conceptualization, supervision, writing – review and editing, software. **Adriana C. Panayi:** validation, writing – review and editing. **Liming Zhong:** investigation and data curation. **Lin Luo:** methodology, resources, and funding acquisition. **Ziyu Chen:** conceptualization, supervision, project administration, writing – review and editing, funding acquisition. **Mengfan Wu:** conceptualization, project administration, supervision, writing – review and editing, funding acquisition.

## Ethics Statement

Ethical approval and informed consent were not required for this study because it was a systematic review and meta‐analysis based exclusively on previously published studies. All authors have read and approved the final version of the manuscript. Mengfan Wu had full access to all data in the study and takes complete responsibility for the integrity of the data and the accuracy of the data analysis.

## Conflicts of Interest

The authors declare no conflicts of interest.

## Transparency Statement

Mengfan Wu affirms that this manuscript is an honest, accurate, and transparent account of the study being reported; that no important aspects of the study have been omitted; and that any discrepancies from the study as planned have been explained.

## Supporting information

Supporting File 1

Supporting File 2

Supporting File 3

Supporting File 4

## Data Availability

The data that support the findings of this study are available from the corresponding author upon reasonable request.
