# Supplementary material for: Negative Pressure Wound Therapy for the Prevention of Wound Complications After Hepatopancreatobiliary Surgery: A Systematic Review and Meta‐Analysis
Source: Health Sci Rep. 2026 Jul 4;9(7):e72749. doi: 10.1002/hsr2.72749 (PMC13332860; doi:10.1002/hsr2.72749)
Supplement: Supplementary file 1 — Supporting File 1 [file HSR2-9-e72749-s003.docx]

**Supplementary Materials 1. Search Strategy.**

Databases to Search:

- PubMed
- Cochrane Library
- Embase
- Web of Science
- Scopus

The search was conducted from inception to Aug 17, 2024.

Keywords:

1. General NPWT Terms:

- Negative-Pressure Wound Therapy
- NPWT
- Negative Pressure Therapy
- Negative Pressure Dressing
- Vacuum-Assisted Closure
- Vacuum sealing drainage
- Vacuum
- VAC
- VAC therapy
- Wound Vac
- Subatmospheric Pressure Therapy
- Sealed Wound Suction
- Topical Negative Pressure Therapy
- TNP Therapy

2. Hepatopancreatobiliary Surgery Terms:

- Hepatopancreatobiliary surgery
- HPB surgery
- Liver surgery
- Hepatic surgery
- Pancreatic surgery
- Biliary surgery
- Cholecystectomy
- Pancreatectomy
- Hepatectomy
- Whipple procedure
- Bile duct surgery
- Pancreaticoduodenectomy
- Hepatic resection
- Pancreatic resection
- Gallbladder surgery
- Liver transplantation
- Hepaticojejunostomy
- Pancreaticojejunostomy
- Hepatic lobectomy
- Segmentectomy
- Cystic duct excision

**Search Strings:**

1. PubMed:

("Negative-Pressure Wound Therapy"[Mesh] OR "negative pressure wound therapy" OR NPWT OR "negative pressure therapy" OR "negative pressure dressing" OR "vacuum-assisted closure" OR "vacuum sealing drainage" OR vacuum OR VAC OR "VAC therapy" OR "wound vac" OR "subatmospheric pressure therapy" OR "sealed wound suction" OR "topical negative pressure therapy" OR "TNP therapy")

AND

("Hepatopancreatobiliary Surgery"[Mesh] OR "hepatopancreatobiliary surgery" OR "HPB surgery" OR "liver surgery" OR "hepatic surgery" OR "pancreatic surgery" OR "biliary surgery" OR cholecystectomy OR pancreatectomy OR hepatectomy OR "Whipple procedure" OR "bile duct surgery" OR pancreaticoduodenectomy OR "hepatic resection" OR "pancreatic resection" OR "gallbladder surgery" OR "liver transplantation" OR hepaticojejunostomy OR pancreaticojejunostomy OR "hepatic lobectomy" OR segmentectomy OR "cystic duct excision")

1. Cochrane Library:

("Negative-Pressure Wound Therapy" OR "negative pressure wound therapy" OR NPWT OR "negative pressure therapy" OR "negative pressure dressing" OR "vacuum-assisted closure" OR "vacuum sealing drainage" OR vacuum OR VAC OR "VAC therapy" OR "wound vac" OR "subatmospheric pressure therapy" OR "sealed wound suction" OR "topical negative pressure therapy" OR "TNP therapy")

AND

("Hepatopancreatobiliary Surgery" OR "hepatopancreatobiliary surgery" OR "HPB surgery" OR "liver surgery" OR "hepatic surgery" OR "pancreatic surgery" OR "biliary surgery" OR cholecystectomy OR pancreatectomy OR hepatectomy OR "Whipple procedure" OR "bile duct surgery" OR pancreaticoduodenectomy OR "hepatic resection" OR "pancreatic resection" OR "gallbladder surgery" OR "liver transplantation" OR hepaticojejunostomy OR pancreaticojejunostomy OR "hepatic lobectomy" OR segmentectomy OR "cystic duct excision")

1. Embase:

('negative-pressure wound therapy' OR 'negative pressure wound therapy' OR NPWT OR 'negative pressure therapy' OR 'negative pressure dressing' OR 'vacuum-assisted closure' OR 'vacuum sealing drainage' OR vacuum OR VAC OR 'VAC therapy' OR 'wound vac' OR 'subatmospheric pressure therapy' OR 'sealed wound suction' OR 'topical negative pressure therapy' OR 'TNP therapy')

AND

('hepatopancreatobiliary surgery' OR 'HPB surgery' OR 'liver surgery' OR 'hepatic surgery' OR 'pancreatic surgery' OR 'biliary surgery' OR cholecystectomy OR pancreatectomy OR hepatectomy OR 'Whipple procedure' OR 'bile duct surgery' OR pancreaticoduodenectomy OR 'hepatic resection' OR 'pancreatic resection' OR 'gallbladder surgery' OR 'liver transplantation' OR hepaticojejunostomy OR pancreaticojejunostomy OR 'hepatic lobectomy' OR segmentectomy OR 'cystic duct excision')

1. Web of Science:

TS=("Negative-Pressure Wound Therapy" OR "negative pressure wound therapy" OR NPWT OR "negative pressure therapy" OR "negative pressure dressing" OR "vacuum-assisted closure" OR "vacuum sealing drainage" OR vacuum OR VAC OR "VAC therapy" OR "wound vac" OR "subatmospheric pressure therapy" OR "sealed wound suction" OR "topical negative pressure therapy" OR "TNP therapy")

AND

TS=("Hepatopancreatobiliary Surgery" OR "hepatopancreatobiliary surgery" OR "HPB surgery" OR "liver surgery" OR "hepatic surgery" OR "pancreatic surgery" OR "biliary surgery" OR cholecystectomy OR pancreatectomy OR hepatectomy OR "Whipple procedure" OR "bile duct surgery" OR pancreaticoduodenectomy OR "hepatic resection" OR "pancreatic resection" OR "gallbladder surgery" OR "liver transplantation" OR hepaticojejunostomy OR pancreaticojejunostomy OR "hepatic lobectomy" OR segmentectomy OR "cystic duct excision")

1. Scopus:

(TITLE-ABS-KEY("Negative-Pressure Wound Therapy" OR "negative pressure wound therapy" OR NPWT OR "negative pressure therapy" OR "negative pressure dressing" OR "vacuum-assisted closure" OR "vacuum sealing drainage" OR vacuum OR VAC OR "VAC therapy" OR "wound vac" OR "subatmospheric pressure therapy" OR "sealed wound suction" OR "topical negative pressure therapy" OR "TNP therapy"))

AND

(TITLE-ABS-KEY("Hepatopancreatobiliary Surgery" OR "hepatopancreatobiliary surgery" OR "HPB surgery" OR "liver surgery" OR "hepatic surgery" OR "pancreatic surgery" OR "biliary surgery" OR cholecystectomy OR pancreatectomy OR hepatectomy OR "Whipple procedure" OR "bile duct surgery" OR pancreaticoduodenectomy OR "hepatic resection" OR "pancreatic resection" OR "gallbladder surgery" OR "liver transplantation" OR hepaticojejunostomy OR pancreaticojejunostomy OR "hepatic lobectomy" OR segmentectomy OR "cystic duct excision"))
