# Supplementary material for: Negative Pressure Wound Therapy for the Prevention of Wound Complications After Hepatopancreatobiliary Surgery: A Systematic Review and Meta‐Analysis
Source: Health Sci Rep. 2026 Jul 4;9(7):e72749. doi: 10.1002/hsr2.72749 (PMC13332860; doi:10.1002/hsr2.72749)

**Supplementary Materials 2. Risk of bias.**

| **Study** | **Selection** | | | | **Comparability** | **Outcome** | | | **Total score** |
| --- | --- | --- | --- | --- | --- | --- | --- | --- | --- |
|  | Representative-ness of the exposed cohort | Selection of the non exposed cohort | Ascertainment of exposure | Demonstration that outcome of interest was not present at start of study | Comparability of cohorts on the basis of the design or analysis | Assessment of outcome | Was follow-up long enough for outcomes to occur | Adequacy of follow up of cohorts |  |
| Burkhart 2017 | 🟑 | 🟑 | 🟑 | 🟑 | 🟑🟑 | 🟑 | 🟑 | 🟑 | 9/9 |
| Greene 2023 | 🟑 | 🟑 | 🟑 | 🟑 | 🟑 | 🟑 | 🟑 | 🟑 | 8/9 |
| Gupta 2017 | 🟑 | 🟑 |  | 🟑 | 🟑 | 🟑 |  |  | 5/9 |
| Lawrence2019 | 🟑 | 🟑 | 🟑 | 🟑 | 🟑 | 🟑 | 🟑 | 🟑 | 8/9 |


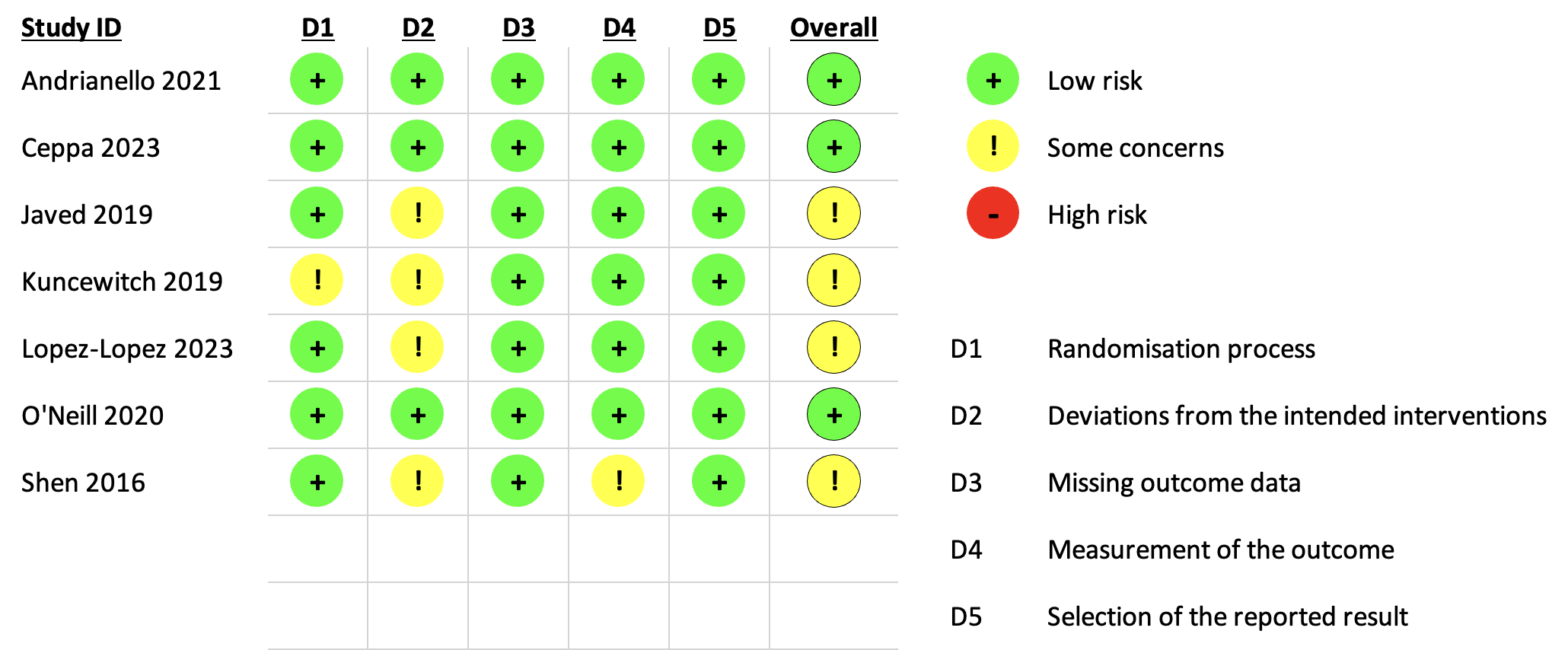

Supplement: Supplementary file 2 — Supporting File 2 [file HSR2-9-e72749-s001.docx]
