# Supplementary material for: Negative Pressure Wound Therapy for the Prevention of Wound Complications After Hepatopancreatobiliary Surgery: A Systematic Review and Meta‐Analysis
Source: Health Sci Rep. 2026 Jul 4;9(7):e72749. doi: 10.1002/hsr2.72749 (PMC13332860; doi:10.1002/hsr2.72749)
Supplement: Supplementary file 3 — Supporting File 3 [file HSR2-9-e72749-s002.docx]

**Supplementary Materials 3. Publication bias.**

1. **Funnel plot.**

**
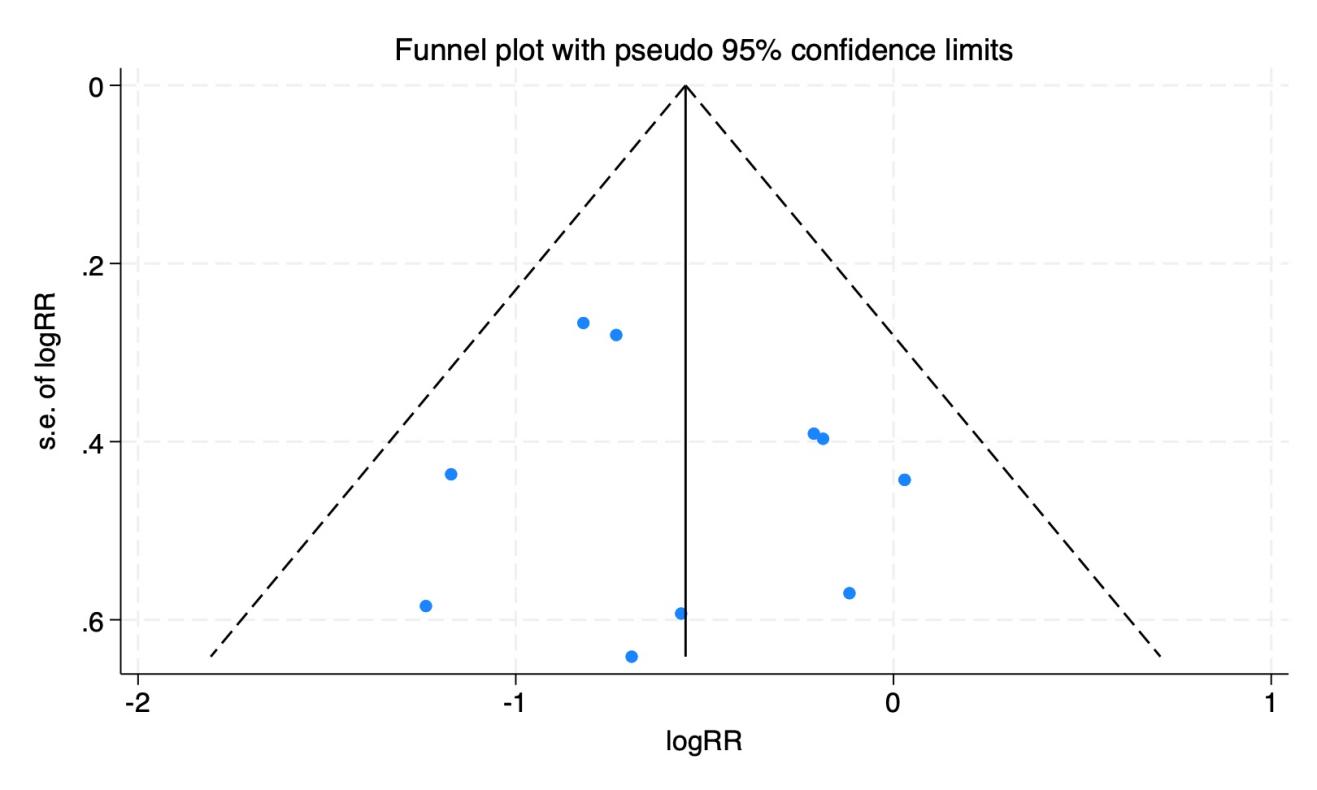
**

1. **Egger’s test.**


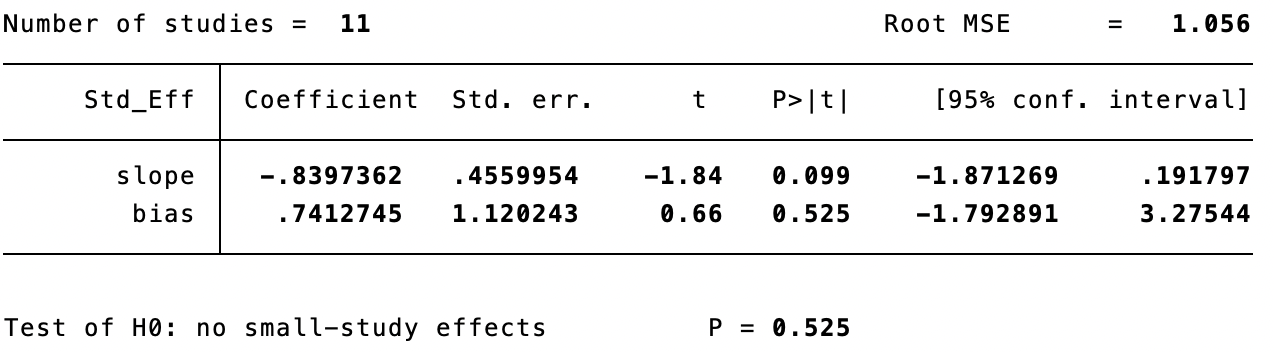


Egger’s regression test did not detect significant publication bias.

1. **Trim-and-fill analysis**


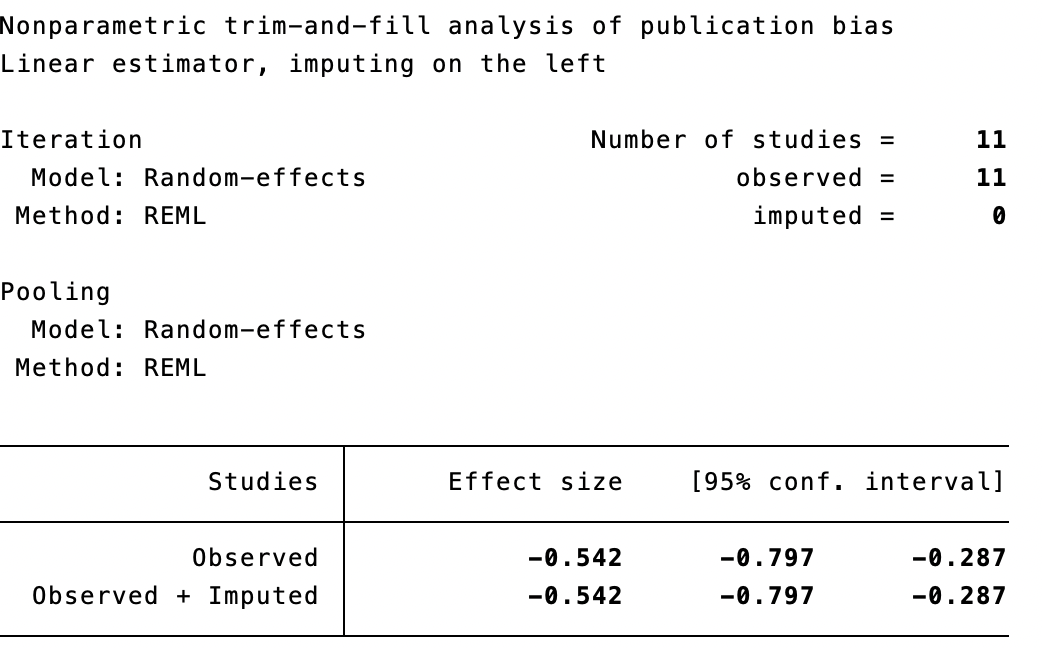


Nonparametric trim-and-fill analysis identified no potentially missing studies, and the pooled effect estimate remained unchanged after adjustment, suggesting relative robustness of the primary findings.
