# Supplementary material for: Negative Pressure Wound Therapy for the Prevention of Wound Complications After Hepatopancreatobiliary Surgery: A Systematic Review and Meta‐Analysis
Source: Health Sci Rep. 2026 Jul 4;9(7):e72749. doi: 10.1002/hsr2.72749 (PMC13332860; doi:10.1002/hsr2.72749)
Supplement: Supplementary file 4 — Supporting File 4 [file HSR2-9-e72749-s004.docx]

**Supplementary Materials 4.**

1. **GRADE assessment.**

| **Outcome** | **No. of Studies** | **Participants** | **Study Design** | **Risk of Bias** | **Inconsistency** | **Indirectness** | **Imprecision** | **Publication Bias** | **Relative Effect (RR, 95% CI)** | **Certainty of Evidence** |
| --- | --- | --- | --- | --- | --- | --- | --- | --- | --- | --- |
| Overall SSI | 11 | 1566 | RCT + observational | Serious | Not serious | Serious | Not serious | Undetected | 0.58 (0.45–0.75) | LOW |
| Superficial SSI | 8 | 889 | RCT + observational | Serious | Serious | Serious | Serious | Undetected | 0.64 (0.42–0.99) | VERY LOW |
| Pancreatectomy subgroup SSI | 10 | 1377 | RCT + observational | Serious | Not serious | Serious | Not serious | Undetected | 0.61 (0.45–0.82) | LOW |

1. **Statistical Software**

Meta-analyses were performed using Review Manager (RevMan) version 5.3. Additional publication bias analyses, including Egger’s regression test and trim-and-fill analysis, were performed using Stata version 19.0.

1. **Data Extraction Process**

Two investigators (XM and LL) independently extracted study data using a standardized data extraction form developed prior to study initiation.

Extracted variables included:

1. Study characteristics (author, year, country, study design)

2. Patient demographics and sample size

3. Surgical procedure type

4. NPWT device type and treatment protocol

5. Comparator dressing type

6. Outcome measures, including surgical site infection subtypes and secondary wound complications

7. Follow-up duration

Disagreements between reviewers were resolved through discussion and consensus with a third investigator (MW) when necessary.
